# The impact of transsphenoidal surgery on pituitary function in patients with non-functioning macroadenomas

**DOI:** 10.1007/s12020-023-03400-z

**Published:** 2023-05-24

**Authors:** Maria Mavromati, Thomas Mavrakanas, François R. Jornayvaz, Karl Schaller, Aikaterini Fitsiori, Maria I. Vargas, Johannes A. Lobrinus, Doron Merkler, Kristof Egervari, Jacques Philippe, Sophie Leboulleux, Shahan Momjian

**Affiliations:** 1 Service of Endocrinology, Diabetes, Nutrition and Therapeutic Patient Education, WHO Collaborating Center, Geneva University Hospital, Geneva University, Geneva, Switzerland; 2 Division of Nephrology, McGill University Health Center, McGill University, Montreal, QC Canada; 3 Service of Neurosurgery, Geneva University Hospital, Geneva University, Geneva, Switzerland; 4 Service of Neurodiagnostic, Division of Neuroradiology, Geneva University Hospital, Geneva University, Geneva, Switzerland; 5 Service of Clinical Pathology, Geneva University Hospital, Geneva, Switzerland; 6 Geneva University, Geneva, Switzerland

**Keywords:** NFPAs, Transsphenoidal surgery, Pituitary function, Hypopituitarism

## Abstract

**Purpose:**

Transsphenoidal surgery for non-functioning pituitary adenomas (NFPAs) can alter pituitary function. We assessed the rates of improvement and deterioration of pituitary function by axis and searched for predictive factors of these outcomes.

**Methods:**

We reviewed consecutive medical files from patients having had transsphenoidal surgery for NFPA between 2004 and 2018. Pituitary functions and MRI imaging were analyzed prior and after surgery. The occurrence of recovery and new deficit were documented per axis. Prognostic factors of hormonal recovery and new deficits were searched.

**Results:**

Among 137 patients analyzed, median tumor size of the NFPA was 24.8 mm and 58.4% of patients presented visual impairment. Before surgery, 91 patients (67%) had at least one abnormal pituitary axis (hypogonadism: 62.4%; hypothyroidism: 41%, adrenal insufficiency: 30.8%, growth hormone deficiency: 29.9%; increased prolactin: 50.8%). Following surgery, the recovery rate of pituitary deficiency of one axis or more was 46% and the rate of new pituitary deficiency was 10%. Rates of LH-FSH, TSH, ACTH and GH deficiency recovery were 35.7%, 30.4%, 15.4%, and 45.5% respectively. Rates of new LH-FSH, TSH, ACTH and GH deficiencies were 8.3%, 1.6%, 9.2% and 5.1% respectively. Altogether, 24.6% of patients had a global pituitary function improvement and only 7% had pituitary function worsening after surgery. Male patients and patients with hyperprolactinemia upon diagnosis were more likely to experience pituitary function recovery. No prognostic factors for the risk of new deficiencies were identified.

**Conclusion:**

In a real-life cohort of patients with NFPAs, recovery of hypopituitarism after surgery is more frequent than the occurrence of new deficiencies. Hence, hypopituitarism could be considered a relative indication for surgery in patients with NFPAs.

## Introduction

Pituitary adenomas are the most common tumors of the sella turcica and are of benign nature. Their incidence in autopsy series reaches 10% of the population. Their prevalence ranges in-between 78 and 94 cases per 100,000 habitants in recent studies, showing that these tumors are not as rare as they were believed to be [1, 2]. Non-functioning pituitary adenomas (NFPAs), defined by the absence of clinical and biological evidence of hormonal secretion, represent 25–40% of all pituitary adenomas [3–5] They are the second most common subtype of pituitary adenomas with prolactinomas being the most prevalent (40–55%). The other subtypes are less frequent, consisting in GH-secreting adenomas in 10% of the cases, ACTH-secreting adenomas in 1–5% of the cases and TSH-secreting adenomas in less than 1% of the cases [6].

Immunohistochemistry of NFPAs usually shows gonadotropin expression (68%), or no hormonal expression at all (null cell adenomas, 27%), while GH, ACTH, TSH or even prolactin expression is quite rare (silent adenomas, 5%) [7, 8]. With routine use of immunohistochemistry for transcription factors, as recommended by the 2022 World Health Organization (WHO) classification for pituitary tumors, pituitary adenomas are distinguished in PIT1-lineage, TPIT-lineage and SF1-lineage pituitary neuroendocrine tumors (PitNETs), with gonadotroph tumors included in the latter category and null cell tumors having no distinct cell lineage, thus, being a diagnosis of exclusion [9].

Based on their size, pituitary adenomas are classified as microadenomas (≤10 mm) or macroadenomas (>10 mm). Unlike non-functioning (NF) microadenomas, which will rarely grow during follow-up, and among which only 5% will exceed 10 mm in diameter, NF macroadenomas seem to have a higher growth potential. Indeed, 25–50% of these tumors will progress during a median follow-up of 2–7 years [10].

In the absence of surgical treatment, the risk of developing new hormone deficiencies in patients with pituitary macroadenomas is estimated to be 12% per year [11]. For NF macroadenomas, surgery is mainly indicated in case of visual impairment. Guidelines from the Endocrine Society suggest that surgery may also be considered in case of hypopituitarism despite the absence of visual impairment, but the volume of evidence is quite limited [6]. The impact of surgery on anterior pituitary function is not yet very well established and risks of new postoperative pituitary deficiencies and recovery after surgery vary among studies [10]. Serious complications related to transsphenoidal surgery are rare (mortality ≤1% and other non-lethal serious complications ≤5%), but cannot be neglected [12]. It is also necessary to take into account the risk of apoplexy of unoperated pituitary adenomas, which is however low and estimated to be of 1% per year [11]. Thus, in the absence of visual impairment, the utility of surgery is controversial, and the decision must be individualized by weighing potential benefits of the intervention and risk of complications.

The objectives of our study were to evaluate the impact of transsphenoidal surgery on anterior pituitary function in patients with NF macroadenomas and to assess factors predicting postoperative recovery and new deficiencies.

## Materials and methods

### Population and data

**Fig. 1:**
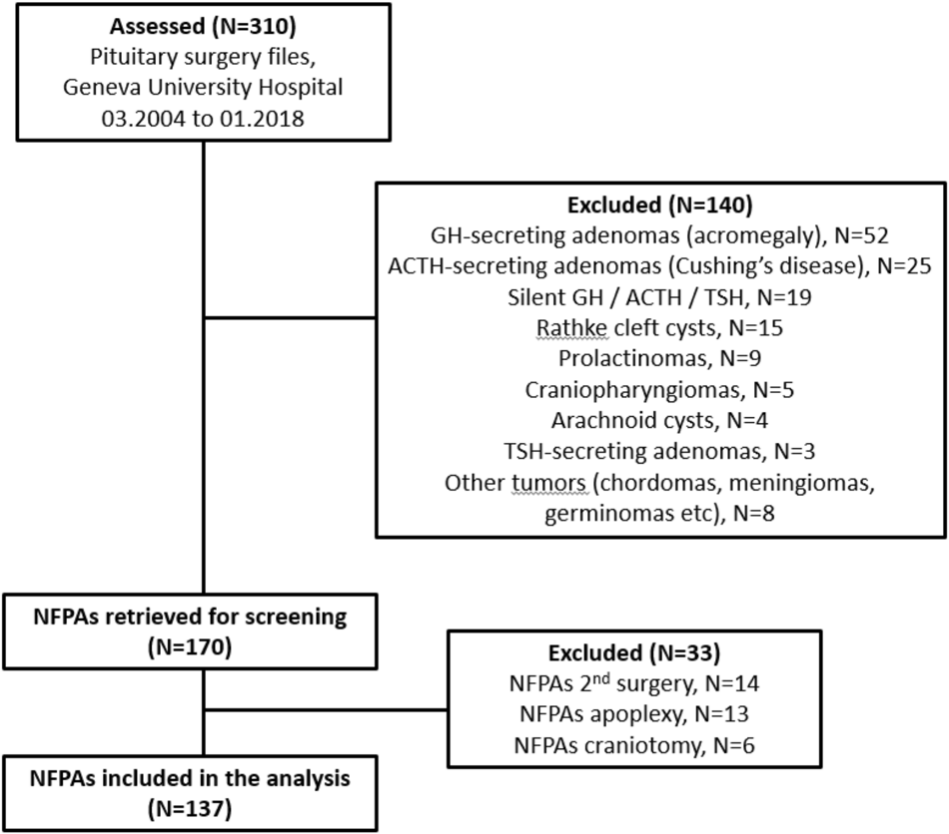
Flow-chart of pituitary surgeries screened and selected for inclusion in the analysis

We retrospectively reviewed files from 310 consecutive pituitary surgeries performed in Geneva University Hospital, a tertiary center, from March 2004 until January 2018 and selected patients having had surgery for a NF pituitary macroadenoma, on the basis of clinical and biochemical data as well as pathological analysis of the tumor. Clinically functioning adenomas, Rathke cleft cysts, apoplexy, microadenomas, other types of sellar tumors (e.g., craniopharyngiomas), transcranial approach surgery, second surgery, patients with previous radiotherapy, as well as silent ACTH-secreting and GH-secreting adenomas were excluded (Fig. 1). Finally, the analysis included NF pituitary macroadenomas at the time of their first surgical treatment. We documented time, age, symptoms and signs upon diagnosis and surgery, duration of follow-up until surgery, visual field defects and visual acuity as well as reasons for surgery. The study was approved by the Swiss Ethics Committee in compliance with the Declaration of Helsinki; a waiver of informed consent was granted, as the study was determined to involve no risk to the subjects included by using existing medical file information.

### Assessment of tumor size

Tumor size was evaluated on gadolinium-enhanced magnetic resonance imaging (MRI), performed 0–3 months before surgery, as well as 3–6 months postoperatively. MRI files were all reviewed in 2-dimension T1 sequences after gadolinium injection and adenomas were measured in 3 diameters (cranio-caudal, antero-posterior and transverse). Patients were classified, before and after surgery, according to the maximal diameter (Dmax) and according to the average diameter (Dav), calculated as the average of 3 diameters, in 4 size groups (S1: 10–19 mm, S2: 20–29 mm, S3: 30–39 mm and S4: ≥40 mm). Finally, we classified patients according to percentage of decrease of the average diameter with surgery, in groups 0 to 3 (0: ≥80% decrease, 1: 50–79, 2: 20–49 and 3: <20% decrease).

### Evaluation of hormonal results

We retrieved and collected the results of hormonal workup performed up to 6 months prior to surgery, as well as 3–6 months postoperatively. Tests had been performed either in Geneva University Hospital, or in private laboratories, and had been organized by the treating physician. Diagnosis of hypopituitarism in our study was based on biologic tests according to normative values suggested by each laboratory, as well as symptoms related to hormonal deficiency, and patients were classified as having a normal or an impaired function, per axis. Percentages of hormonal deficiencies were calculated for patients with available data and not for the whole cohort.

Evaluation of GH deficiency was based on IGF-1 levels, and classification of patients was performed according to age-related normative values suggested by each laboratory.

Assessment of central adrenal insufficiency was based on basal plasma cortisol levels as well as a standard-dose (250 μg) ACTH stimulation test. Patients were classified in five groups as follows: “normal” (if basal cortisol levels were >400 nmol/l or if the level rose >500 nmol/l after ACTH stimulation test), “probably normal” (if dynamic test had not been performed but basal cortisol levels were >350 nmol/l), “central adrenal insufficiency” (if 8 a.m. plasma cortisol levels were <70 nmol/l or <500 nmol/l after dynamic testing) and “possible insufficiency” (if dynamic test had not been performed, 8 a.m. plasma cortisol levels were 70–350 nmol/l, and the patient was receiving glucocorticoid replacement because of symptoms related to possible central adrenal insufficiency). Confounding factors, such as ongoing oral estrogen use, were documented and those patients were thus not classified into the above-mentioned categories. Finally, several patients had an incomplete assessment and could not be classified in the categories described above. On the basis of this classification, two types of analysis were carried out for the ACTH axis evaluation, a strict analysis, comparing only “normal” patients to those with well-documented “central adrenal insufficiency”, in line with cut-offs suggested in the 2016 Endocrine Society guidelines for hypopituitarism, as well as a simplified analysis, where “normal” and “probably normal” patients were considered together and compared to patients with “central adrenal insufficiency” and “possible insufficiency” who were also considered together [13].

Assessment of central hypogonadism was based on gender and age. In women of childbearing age, diagnosis of central hypogonadism was based on the presence of oligomenorrhea or amenorrhea, low serum estradiol levels and normal or low FSH/LH levels. In women older than 60 years of age, FSH/LH values at pre-menopausal levels, according to normative values suggested by each laboratory, were sufficient for the diagnosis of central hypogonadism. In men, central hypogonadism was defined by the presence of low total testosterone levels and normal or low LH, together with symptoms or signs of testosterone deficiency. Confounding factors to the evaluation of the gonadal axis were also documented and patients taking hormonal substitution or contraception were not classified.

Diagnosis of central hypothyroidism was based on a low free-T4 together with low or normal TSH. Postoperatively, evaluation was only taken into account if tests had been performed after discontinuation of thyroid hormone replacement if started after surgery. Patients on levothyroxine for primary hypothyroidism were considered as non-assessable.

Patients were classified on the basis of prolactin levels (elevated, normal or low) before and after surgery. To eliminate the bias of the impact of hyperprolactinemia on the gonadal axis, a second analysis of the latter was performed, after excluding patients with hyperprolactinemia.

In order to evaluate clinically relevant global pituitary function improvement or worsening, we performed an additional analysis after excluding cases of GH deficiency (in the absence of routinely performed dynamic testing) as well as cases of hypogonadism in postmenopausal women. Pituitary function was considered improved if at least one axis had improved with a total number of hormonal deficiencies lower than preoperatively. Pituitary function was considered worse if there was at least one new deficiency and a total number of hormonal deficiencies higher than preoperatively.

### Surgery

Surgery was performed using a microscopic, endoscope-assisted, trans-septal transsphenoidal approach, by the same experienced surgeon. If patients had postoperative basal cortisol levels below 500 nmol/l on day 4, they were then maintained on 5 mg of prednisone per day until a proper biologic evaluation could take place.

### Statistical analysis

Baseline characteristics were reported as mean ± standard deviation, median (interquartile range), or number (percentage), as appropriate. To identify potential factors associated with new hormone deficiencies or the recovery from a previous hormonal deficiency, logistic regression analysis was performed. Five predictors (age per 5 years, gender, tumor size per 5 mm, ≥50% reduction in tumor size after surgery and presence of hyperprolactinemia upon diagnosis) were examined at a univariate level. For the outcome of recovery from a previous hormonal deficiency, predictors with a univariate *p* value < 0.20 were included in the multivariate model. Statistical analyses were performed in SPSS version X and in Stata version 17.0 SE. Any *p* value < 0.05 was considered significant.

## Results

### Patients’ characteristics

Patients’ characteristics are summarized in Table 1. Altogether, 310 pituitary surgery files performed between March 2004 and January 2018 were reviewed, of which 137 cases of non-functioning pituitary macroadenomas were selected. The cohort included 56 women (40.9%) and 81 men (59.1%). Median age at diagnosis was 59 years (range: 28–85) and median age at the time of surgery 60 years (range: 28–86). There were no cases of diabetes insipidus upon diagnosis nor after surgery (3 and 6 months postoperatively). Median time of follow up until surgery was 12.7 months (range: 3–360), while 75% of patients had surgery performed within 8 months from diagnosis and only 14.7% had surgery later than 24 months from diagnosis. On ophthalmologic evaluation following diagnosis (visual field and visual acuity examination), 80 patients (58.4%) had visual impairment. Indication for surgery was visual optic nerve compression on MRI with or without visual impairment in most cases (89.8%, *N* = 123), while 6.6% (*N* = 9) had surgery due to tumor growth during follow-up, and only 3.6% (*N* = 5) for other reasons (hypopituitarism, patient’s preference).

**Table 1:** Patients’ characteristics

Patients’ characteristics	All patients (*N* = 137)
Female/Male, *N* (%)	56 (40.9%)/81 (59.1%)
Age at diagnosis, median in years (range)	59 (28–85)
Age at surgery, median in years (range)	60 (28–86)
Time of follow-up till surgery, median in months (range)	12.7 (3–360)
Reasons leading to diagnosis, *N* (%)	
Incidentaloma	55 (40.1%)
Visual impairment	45 (32.8%)
Symptoms of hormonal dysfunction	23 (16.8%)
Headaches	14 (10.2%)
Visual impairment upon diagnosis, *N* (%)	
Present	80 (58.4%)
Absent	57 (41.6%)
Indication for surgery, *N* (%)	
Visual impairment or optic nerve compression	123 (89.8%)
Tumor growth during follow-up	9 (6.6%)
Other reasons (patient’s preference, hypopituitarism, etc.)	5 (3.6)
Tumor size before surgery by maximal diameter (Dmax), *N* = 137	
S1: 10 ≤ Dmax < 20 mm	35 (25.5%)
S2: 20 ≤ Dmax < 30 mm	66 (48.2%)
S3: 30 ≤ Dmax < 40 mm	26 (19%)
S4: Dmax ≥ 40 mm	10 (7.3%)
Tumor size before surgery by average diameter (Dav), *N* = 137	
S1: Dav < 20 mm	55 (40.1%)
S2: 20 ≤ Dav < 30 mm	67 (48.9%)
S3: 30 ≤ Dav < 40 mm	13 (9.5%)
S4: Dav ≥ 40 mm	2 (1.5%)
Tumor size after surgery by average diameter (Dav), *N* = 137	
S0: no residual tumor	50 (36.5%)
S1: Dav < 10 mm	33 (24.1%)
S2: 10 ≤ Dav < 20 mm	30 (21.9%)
S3: Dav ≥ 20 mm	13 (9.5%)
No data	11 (8%)
Average diameter decrease with surgery (Dav), *N* = 137	
S0: >80%	52 (38%)
S1: 50–79%	41 (29.9%)
S2: 20–49%	20 (14.6%)
S3: <20%	12 (8.8%)
No data	12 (8.8%)

### Tumor size

Classification of patients according to tumor size before and after surgery is also summarized in Table 1. At the time of surgery, median maximal tumor diameter was 24.8 mm (range: 10–50). In 73.7% of cases (*N* = 101), Dmax was <30 mm (89% with Dav <30 mm). On MRI performed 3–6 months after surgery, 36.5% of patients (*N* = 50) had no residual tumor, 24.1% (*N* = 33) had a residual tumor of <10 mm, while 9.5% (*N* = 13) had a residual tumor exceeding 20 mm of average diameter. Overall, postoperatively, 38% of patients (*N* = 52) had Dav decrease of 80% or more, 29.9% (*N* = 41) had a 50–79% decrease and 23.4% (*N* = 32) had <50% decrease.

### Evaluation of the anterior pituitary hormonal axis before and after surgery

**Fig. 2:**
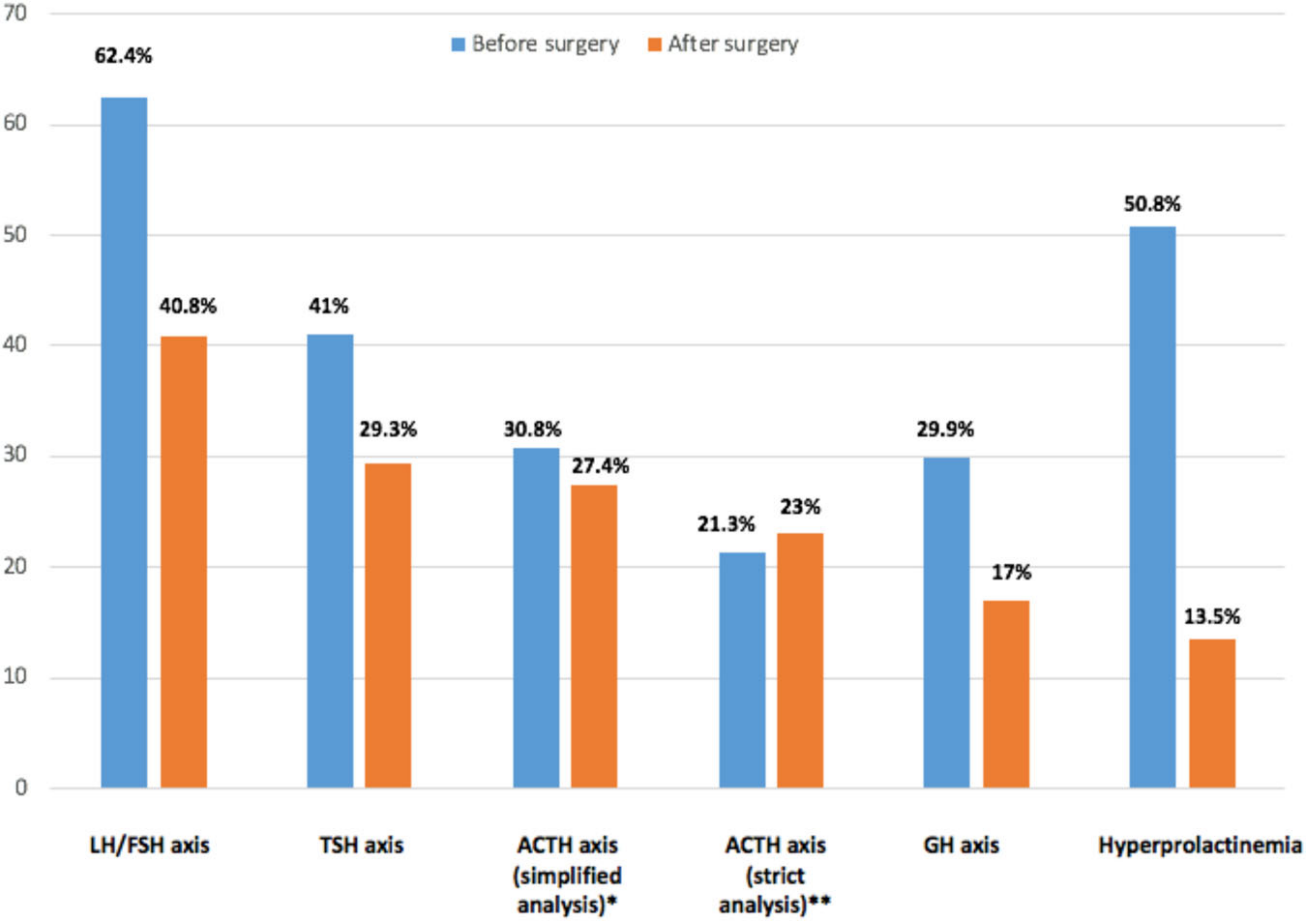
Rates of hormonal insufficiencies per axis and hyperprolactinemia, before and after surgery; patients with data before or after surgery. *Simplified analysis for the ACTH axis: patients with morning cortisol levels >350 nmol/l or >500 nmol/l after ACTH stimulation test were considered as normal, patients with morning cortisol levels <70 nmol/l or <500 nmol/l after dynamic testing as well as patients with morning cortisol levels 70–350 nmol/l but who were receiving glucocorticoid replacement because of symptoms related to possible adrenal insufficiency, were considered as having central adrenal insufficiency. **Strict analysis for the ACTH axis: only patients with morning cortisol levels >400 nmol/l or >500 nmol/l after ACTH stimulation test were considered as normal, only patients with morning cortisol levels <70 nmol/l or <500 nmol/l after dynamic testing were considered as having central adrenal insufficiency

Before surgery, central hypogonadism was detected in 62.4% of patients (31.5% of which were postmenopausal women), central hypothyroidism in 41%, central adrenal insufficiency in 30.8% (21.3% with the strict analysis), and low IGF-1 in 29.9% of patients (Table 2 and Fig. 2). High prolactin levels were detected in 50.8% of patients. After surgery, central hypogonadism was detected in 40.8% of patients (30% of which were postmenopausal women), central hypothyroidism in 29.3%, central adrenal insufficiency in 27.4% (22.7% with the strict analysis), and low IGF-1 in 17% of patients (Table 2 and Fig. 2). High prolactin level was detected in 13.5% of patients.

**Table 2:** Classification of patients according to hormonal workups by axis, before and after surgery

	Prior to surgery	After surgery
LH/FSH		
Number of patients analyzed	117^a^	98^b^
Normal, *n*(%)	44 (37.6%)	58 (59.2%)
Insufficiency, *n*(%)	73 (62.4%)	40 (40.8%)
Insufficiency in postmenopausal women, *n*(%)	23 (19.7%)	12 (12.2%)
TSH		
Number of patients analyzed	122^c^	116^d^
Normal, *n*(%)	72 (59%)	82 (70.7%)
Insufficiency, *n*(%)	50 (41%)	34 (29.3%)
ACTH		
Number of patients analyzed	131^e^	124^f^
Normal, *n*(%)	59 (45%)	75 (60.5%)
Probably normal, *n*(%)	13 (9.9%)	7 (5.6%)
Insufficiency, *n*(%)	16 (12.2%)	22 (17.7%)
Possible insufficiency, *n*(%)	16 (12.2%)	9 (7.3%)
Inconclusive data, *n*(%)	5 (3.8%)	4 (3.2%)
Confounding factors, *n*(%)	22 (16.8%)	7 (5.6%)
ACTH (simplified analysis)		
Number of patients analyzed	104^e^	113^f^
Normal + Probably normal, *n*(%)	72 (69.2%)	82 (72.6%)
Insufficiency + Possible insufficiency, *n*(%)	32 (30.8%)	31 (27.4%)
ACTH (strict analysis)		
Number of patients analyzed	75^e^	97^f^
Normal, *n*(%)	59 (78.7%)	75 (77.3%)
Insufficiency, *n*(%)	16 (21.3%)	22 (22.7%)
GH		
Number of patients analyzed	117^g^	88^h^
Normal, *n*(%)	82 (70.1%)	73 (83%)
Insufficiency, *n*(%)	35 (29.9%)	15 (17%)
Prolactin		
Number of patients analyzed	130^i^	89^j^
High, *n*(%)	66 (50.8%)	12 (13.5%)
Normal, *n*(%)	63 (48.5%)	75 (84.3%)
Low, *n*(%)	1 (0.8%)	2 (2.2%)

^a^No data in 16 cases, confounding factors in 4 cases

^b^No data in 37 cases, confounding factors in 2 cases

^c^No data in 7 cases, confounding factors in 8 cases

^d^No data in 17 cases, confounding factors in 4 cases

^e^No data in 6 cases

^f^No data in 13 cases

^g^No data in 20 cases

^h^No data in 49 cases

^i^No data in 7 cases

^j^No data in 48 cases

**Fig. 3:**
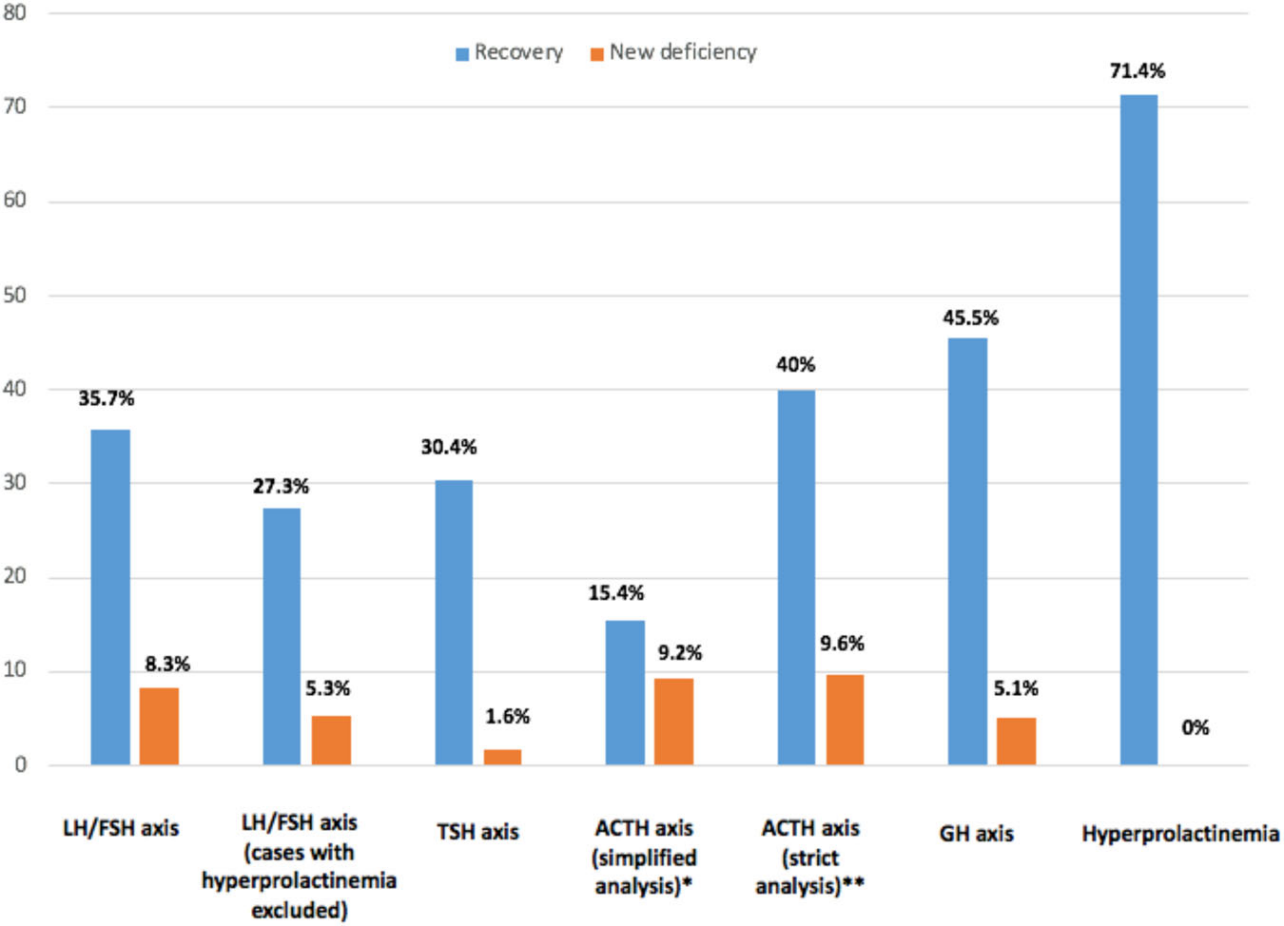
Rates of new deficiencies or recovery after surgery, per axis. *Simplified analysis for the ACTH axis: patients with morning cortisol levels >350 nmol/l or >500 nmol/l after ACTH stimulation test were considered as normal, patients with morning cortisol levels <70 nmol/l or <500 nmol/l after dynamic testing as well as patients with morning cortisol levels 70–350 nmol/l but who were receiving glucocorticoid replacement because of symptoms related to possible adrenal insufficiency, were considered as having central adrenal insufficiency. **Strict analysis for the ACTH axis: only patients with morning cortisol levels >400 nmol/l or >500 nmol/l after ACTH stimulation test were considered as normal, only patients with morning cortisol levels <70 nmol/l or <500 nmol/l after dynamic testing were considered as having central adrenal insufficiency

Among 122 patients with data available before and after surgery, 42 (34.4%) showed recovery of at least one deficiency and 13 (10.6%) experienced the occurrence of one or more new hormonal deficiencies (Fig. 3). Gonadotropin secretory reserve recovered in 35.7% (20/56) of patients (5 of which were postmenopausal women, 25%) while new-onset central hypogonadism occurred in 8.3% (3/36) of patients (none of which were postmenopausal women). Those numbers were 27.3% and 5.3%, respectively for the 41 patients without hyperprolactinemia upon diagnosis. Thyrotropin secretory reserve recovered in 30.4% (14/46) of cases, while new-onset central hypothyroidism occurred in 1.6% (1/62) of patients. With the simplified analysis of the ACTH axis, ACTH axis recovered in 15.4% (4/26) of patients while new-onset central adrenal insufficiency occurred, in 9.2% of patients. With the strict analysis of ACTH-secretion evaluation, ACTH secretory reserve recovered in 40% of them (4/10), while new-onset central adrenal insufficiency occurred in 9.6% of patients (5/52). Recovery of the GH axis occurred in 45.5% (10/22) of patients while new-onset GH deficiency after surgery occurred in 5.1% (3/59). Finally, prolactin levels normalized in 71.4% of cases (30/42), while no cases of new hyperprolactinemia were detected after surgery.

In an additional analysis evaluating global pituitary function change with surgery and taking into account only clinically relevant deficiencies requiring treatment, we found 30 patients with global improvement of pituitary function (24.6%) and 7 with global worsening (5.7%), among 122 with data available before and after surgery in at least one axis.

### Predictors of new deficiencies and recovery after surgery

In the univariate analysis, there was a trend toward increased chances of recovery of at least one deficient hormonal axis in patients with hyperprolactinemia, male gender and older patients which did not reach statistical significance (Table 3). Tumor size was not associated with recovery. In multivariate analysis, hyperprolactinemia and male gender were confirmed to predict postoperative recovery of at least one deficient hormonal axis (Table 3).

**Table 3:** Factors predicting pituitary function improvement and worsening

	Factors predicting recovery of at least 1 hormonal axis (*N* = 42/122)				Factors predicting appearance of at least 1 new hormonal deficiency (*N* = 13/122)	
	Univariate analysis		Multivariate analysis		Univariate analysis	
	OR (95% CI)	*p* value	OR (95% CI)	*p* value	OR (95% CI)	*p* value
Gender (female)	0.51 (0.23–1.15)	0.104	0.27 (0.10–0.71)	0.009	0.997 (0.31–3.25)	0.996
Age (per 5 years)	1.12 (0.98–1.30)	0.106	1.14 (0.98–1.34)	0.095	1.16 (0.92–1.45)	0.214
Tumor size prior to surgery—maximal diameter (per 5 mm)	0.95 (0.75–1.19)	0.637			1.06 (0.75–1.49)	0.749
Average diameter decrease with surgery (at least 50% decrease)	1.07 (0.45–2.56)	0.882			0.79 (0.22–2.77)	0.712
Hyperprolactinemia	2.09 (0.96–4.57)	0.064	3.45 (1.38–8.64)	0.008	1.57 (0.48–5.12)	0.455

On the contrary, in univariate analysis, gender, age, tumor size and hyperprolactinemia, were not found to independently predict the appearance of new hormonal deficiencies after surgery, and thus, a multivariate analysis was not performed (Table 3).

## Discussion

In the absence of threat for the optic pathways, surgery for non-functioning pituitary macroadenoma is not systematically recommended. Nevertheless, if a potential improvement of pituitary function with surgery is aimed at, it would be useful to have prognostic factors of better outcome in order to be able to select patients who could benefit from the surgical excision in the absence of visual impairment.

The impact of hypopituitarism, whether preoperative or postoperative, on patients’ long-term outcomes, must be taken into account in order to assess potential benefits of surgery. Current data suggest higher morbidity and mortality in patients with NF pituitary macroadenoma, and hypopituitarism is found to be a risk factor, regardless of it being the result of surgery or occurring during the simple surveillance of a growing adenoma [14]. A Danish registry-based study on 192 patients who had surgery for a NFPA did not find any increase in mortality (standardized mortality ratio—SMR: 1.21, 95% CI: 0.93–1.59), regardless of the presence of hypopituitarism [15]. On the other hand, the largest study on the topic, based on a Swedish registry including 2795 patients with NFPA (among whom 52% had surgery), found, after follow-up of a median of 7 years, a small but significant increase in mortality (SMR: 1.10; 95% CI: 1.00–1.20). Mortality was significantly higher in patients who were younger than 40 years old (SMR: 2.68, 95% CI: 1.23–5.09), and in women (SMR: 1.29, 95% CI: 1.11–1.48), and was attributed to cerebrovascular and infectious causes [16]. In a British registry-based study including 546 patients who had surgery for NFPA, increased mortality was found, after follow-up of a median of 8 years (SMR: 3.5; 95% CI: 2.8–4.4). In this cohort, the only independent predictor of mortality was age at diagnosis (hazard ratio (HR): 1.1 if >50 years, 95% CI: 1.07–1.13, *p* < 0.001) [17]. Furthermore, hypopituitarism requiring treatment seems to contribute to morbidity of patients with NFPA. Treatment for central adrenal insufficiency for example requires adjustment of glucocorticoids and overtreatment has been associated to increased mortality [18]. In addition, patients with growth hormone deficiency after surgical management for a NFPA and who do not receive treatment with GH, seem to have a higher risk of developing type 2 diabetes (odds ratio (OR): 1.65, 95% CI: 1.06–2.46, *p* = 0.018) [19]. A British population study on 519 patients (90.6% operated, 9.4% observed) with a follow-up of 7 years, showed that central adrenal insufficiency and central hypogonadism were associated with increased mortality (relative risk (RR): 2.26; 95% CI: 1.15–4.47, and 2.56; 95% CI: 1.10–5.96 respectively). In this study, there was a trend toward higher mortality rates with the accumulation of pituitary hormonal deficiencies, and patients with panhypopituitarism had the highest level of risk. Finally, in the same study, glucocorticoid overtreatment and levothyroxine undertreatment were also associated with increased mortality (*p* trend at 0.02 and 0.03, respectively) [20].

Transsphenoidal surgery is performed by a purely endoscopic or by a traditional microscopic approach, or by a combination of the techniques, and prospective studies failed to show superiority of one technique over the other, with complication rates that seem equal [11, 21]. In terms of results on anterior pituitary function, the endoscopic approach appears to be equivalent to traditional microscopic surgery on recent studies [22] while previous reports seemed to support superiority of the endoscopic approach [23].

Hypopituitarism is common in patients with NFPAs and, in our study, central hypogonadism and central hypothyroidism were the most frequently observed deficiencies upon diagnosis (62.4% and 41%, respectively). A retrospective cohort including 246 NFPA patients from France and Belgium reported similar findings at baseline [24]. However, data concerning the impact of transsphenoidal surgery per se on anterior pituitary function are inconsistent, and while the global rate of pituitary function improvement seems to be quite consistent among studies, up to 50%, the risk of new hypopituitarism after surgery is variable, ranging from 1.5 to 22% [10, 11, 24–30].

We reported at least one new pituitary hormonal deficiency after surgery in 9.6% of patients and a 45.5% recovery rate of at least one axis. Global pituitary function improved in 24.6% of patients and worsened in only 5.7%. The impact of surgery was different for each hormonal axis. ACTH secretory reserve was the most fragile (new deficiency in 9.6% of patients after surgery with the strict analysis or 9.2% with the simplified one), while TSH secretory reserve was found to be the most resistant (new deficiency in 1.6% of patients after surgery). Recovery was less frequent for central adrenal insufficiency compared to other hormonal axis only when the simplified criteria were used (15.4%) but reached the highest levels of recovery rate with the strict analysis (40%). The lowest rate of hypopituitarism after surgery described so far is 1.5% and was reported by a German study including 721 NFPAs [27]. Yet, in the same cohort, recovery rate was only 40.8%.

Only a few studies have studied factors predicting new hypopituitarism or recovery after transsphenoidal surgery for NFPAs. Smaller tumors, younger age upon diagnosis and hyperprolactinemia were factors associated with recovery of pituitary function after surgery [24, 27–30]. In our study, hyperprolactinemia was the strongest predictor of recovery (OR: 3.45, 95% CI: 1.38–8.64, *p* = 0.008), in a multivariate regression analysis model including gender and age. It is difficult to explain the mechanism underlying this association. One reason, suggested by the authors of the French and Belgian cohort [24], could be the fact that pituitary stalk compression, rather than destruction of pituitary cells, is the possible cause of hypopituitarism in patients with hyperprolactinemia, and can thus be reversible with surgery.

As for the risk of new postoperative deficiencies, only a few studies showed higher risk in patients with larger tumors [28, 30]. This finding was not confirmed by our study; indeed, we did not identify any predictive factor of pituitary function worsening with surgery, however, this finding must be interpreted with caution since sample size for this analysis was very small (only 13 patients, among 122 with data available before and after surgery, with at least one new hormonal deficiency).

The main limitations of our study are related to its retrospective design. Missing data were present, especially in hormonal axis considered “non clinically relevant”, as the gonadal axis in women after menopause. Nevertheless, it represents real-life practice and underlines the importance of a rigorous hormonal evaluation. Indeed, we discovered that only 54.8% of patients had a complete workup of ACTH secretory reserve upon diagnosis and 70.8% after surgery; those patients had lower rates of central adrenal insufficiency compared to patients with a less detailed evaluation, thus avoiding the continuation of unnecessary glucocorticoid replacement therapy. Evaluation of GH deficiency by means of serum IGF-1 lacks sensitivity since about 20% of adults with GH deficiency have normal IGF-1 levels, still, dynamic testing is not routinely performed in real-life practice in asymptomatic patients. The assessment of tumor size remains imperfect, especially in the presence of bilateral residual tumors, with the risk of underestimating the result of surgery. Finally, our results come from a tertiary center and should be generalized with caution; less experienced centers may face increased rates of complications and less favorable outcomes.

## Conclusion

Following transsphenoidal surgery for non-functioning pituitary macroadenoma the rate of at least one new postoperative hormonal deficiency was lower than the recovery rate of at least one hormonal axis. The ACTH secretory reserve was the most fragile while TSH secretory reserve seemed to be the most resistant. Men were more likely to recover from a preexisting central hormonal deficiency after surgery, yet, the presence of hyperprolactinemia was found to be the strongest predictor of pituitary function recovery. Based on the encouraging recovery rate along with the relatively low risk of new deficiencies, hypopituitarism due to a NFPA could be considered as a valid relative indication for surgery.
